# The Dual Role of HLA-C in Tolerance and Immunity at the Maternal-Fetal Interface

**DOI:** 10.3389/fimmu.2019.02730

**Published:** 2019-12-09

**Authors:** Henrieta Papúchová, Torsten B. Meissner, Qin Li, Jack L. Strominger, Tamara Tilburgs

**Affiliations:** ^1^Department of Stem Cell and Regenerative Biology, Harvard University, Cambridge, MA, United States; ^2^Department of Surgery, Beth Israel Deaconess Medical Center, Boston, MA, United States; ^3^Division of Immunobiology, Center for Inflammation and Tolerance, Cincinnati Children's Hospital Medical Center, Cincinnati, OH, United States

**Keywords:** human, pregnancy, decidua, regulatory T cells, effector T cells, decidual NK cells, trophoblast, HLA-G

## Abstract

To establish a healthy pregnancy, maternal immune cells must tolerate fetal allo-antigens and remain competent to respond to infections both systemically and in placental tissues. Extravillous trophoblasts (EVT) are the most invasive cells of extra-embryonic origin to invade uterine tissues and express polymorphic Human Leucocyte Antigen-C (HLA-C) of both maternal and paternal origin. Thus, HLA-C is a key molecule that can elicit allogeneic immune responses by maternal T and NK cells and for which maternal-fetal immune tolerance needs to be established. HLA-C is also the only classical MHC molecule expressed by EVT that can present a wide variety of peptides to maternal memory T cells and establish protective immunity. The expression of paternal HLA-C by EVT provides a target for maternal NK and T cells, whereas HLA-C expression levels may influence how this response is shaped. This dual function of HLA-C requires tight transcriptional regulation of its expression to balance induction of tolerance and immunity. Here, we critically review new insights into: (i) the mechanisms controlling expression of HLA-C by EVT, (ii) the mechanisms by which decidual NK cells, effector T cells and regulatory T cells recognize HLA-C allo-antigens, and (iii) immune recognition of pathogen derived antigens in context of HLA-C.

## Highlights

- Expression of HLA-C, HLA-E, and HLA-G by EVT in the absence of HLA-A and HLA-B expression, requires trophoblast specific MHC class I transcriptional regulators.
- The expression of a polymorphic paternally inherited HLA-C antigen by EVT provides a target for maternal NK cells and T cells, the HLA-C cell surface expression levels influence how this response is shaped.
- Maternal CTL responses to fetal HLA-C and minor Histocompatibility Antigens (e.g., HY) are generated by many individuals, but during healthy pregnancy HLA-C mismatches are associated with immune tolerance.
- Multiple types of decidual Treg play a role in mechanisms of fetus-specific and non-specific immune tolerance. Further investigation of the function and specificity of decidual Treg has exceptional therapeutic potential for treatment of a wide variety of inflammatory disorders, including pregnancy complications.
- The balance between the transient dysfunction of decidual CD8^+^ T cells and dNK that are permissive of placental and fetal development, and reversal of this dysfunctional state to provide immunity, is crucial in understanding the ethology of pregnancy complications and prevention of congenital infections.
- Interactions of activating KIR with HLA-C reduces the risk of pregnancy complications, possibly through providing specific immunity to viral and bacterial pathogens.

## Introduction

Human Leukocyte Antigen-C (HLA-C) was first discovered by antigen-antibody analysis in the early 1970s (1), but its history started ~10 million years ago on the precursor of the polygenic and polymorphic segment of human chromosome 6, which encodes the Major Histocompatibility Complex (MHC) molecules. HLA-C was formed by a duplication of the HLA-B gene and HLA-C homologs are only present in chimpanzees, gorillas, bonobos, and humans (2). Like the other classical MHC class Ia molecules HLA-A and HLA-B, HLA-C is a highly polymorphic hetero-trimer consisting of an alpha heavy chain, ß2-microglobulin and a peptide antigen (3, 4). Over 5,600 alleles and 3,400 protein variants have thus far been identified for HLA-C (IMGT/HLA sequence database: http://www.ebi.ac.uk/imgt/hla/stats.html) (5). HLA-C is a major determinant for NK cell activity (6, 7) and in 1983 a first report demonstrated the importance of HLA-C in pregnancy and placentation (8). HLA-C has also been associated with many diseases, including viral infections, cancer, autoimmune diseases and transplant failure (9–11). In this review, we critically discuss new insights into: (i) the mechanisms controlling the expression of HLA-C in the absence of HLA-A and HLA-B expression by placental extravillous trophoblasts (EVT); (ii) the mechanisms by which decidual NK cells, effector T cells and regulatory T cells recognize paternal HLA-C allo-antigens during pregnancy and its relevance in the development of pregnancy complications; and (iii) discuss the relevance of immune recognition of pathogen derived antigens in context of HLA-C to establish placental immunity.

## Regulation of HLA-C Expression by EVT

Whereas nearly all nucleated cell types express the polymorphic MHC class Ia molecules HLA-A, HLA-B and HLA-C, as well as the invariant MHC class Ib molecule HLA-E, EVTs lack expression of HLA-A and HLA-B, while expressing HLA-C and HLA-E. In addition, EVT uniquely express the invariant MHC class Ib molecule HLA-G (12–14). Despite the distinct HLA expression phenotype of EVT, the regulatory mechanisms that prevent HLA-A and HLA-B expression while establishing HLA-C, HLA-E, and HLA-G expression on EVT have not been fully elucidated (15–17). NLRC5 (nucleotide-binding domain, leucine-rich repeat family, CARD domain-containing 5), an important transcriptional regulator of MHC class I genes (18–20), and CIITA, the MHC Class II Trans Activator (21), are not expressed by EVT, suggesting that other MHC regulators must be present to control HLA-C, HLA-E, and HLA-G expression in these cells (16). Regulation of HLA-C cell surface expression depends on transcriptional as well as post-transcriptional regulatory mechanisms and the resulting HLA-C cell surface expression levels were shown to impact CD8^+^ effector T cell (Teff) responses to viral infection as well as allogeneic Teff responses (9, 10, 22).

### Transcriptional Regulation of MHC Class I

**Figure 1:**
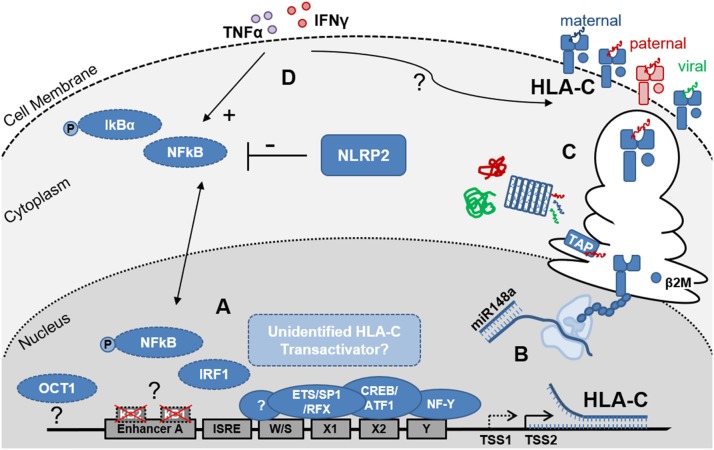
Schematic representation of the factors that influence HLA-C expression levels on EVT. **(A)** Depicts the key elements of the HLA-C promoter region and enhanceosome. Differences between the HLA-C and HLA-A/B promoter regions include the non-functional κB1- and κB2-NFκB binding sites in Enhancer A and the putative OCT1 binding site 800kb upstream of the HLA-C core promoter. EVT do not express the MHC Trans Activators NLRC5 and CIITA, suggesting additional but currently unidentified factors may contribute to regulation of HLA-C transcription. Furthermore, trophoblast specific transcription start sites (TSS) may be present and influence HLA-C mRNA length and stability; **(B)** Post-transcriptional regulation includes the binding of miR148a to the 3′ UTR of the mRNAs of several HLA-C allotypes resulting in reduced HLA-C expression levels; **(C)** Post-translational events including peptide processing by the immunoproteasome and ER-resident Aminopeptidase 1 and 2 (ERAP) as well as peptide loading, by the peptide loading complex (PLC), which is comprised—amongst other subunits- of tapasin and the peptide transporters TAP1 and 2, influence HLA-C protein stability and HLA-C surface expression levels; Allogeneic peptides of paternal origin are depicted in red, maternal peptides in blue and pathogen-derived peptides in green; **(D)** Proinflammatory cytokines including IFNɤ (red) and TNFα (purple) influence HLA-C expression levels through NFκB dependent and independent pathways. Additionally, NOD-like receptors (NLRs) including NLRP2, which is highly expressed by EVT, influence cytokine induced NFκB activation and HLA-C expression.

Both MHC class I and II contain highly conserved cis-regulatory elements in their promoter regions, about 250 base pairs (bp) upstream of the transcriptional start site (23). These include the W/S, X, and Y box motifs that assemble the MHC enhanceosome (24, 25) (Figure 1A). It has recently been shown that the W/S box is crucial for MHC class I transactivation by NLRC5 (24, 26), but no DNA-binding protein of the W/S box has been identified thus far. In the absence of a known DNA binding domain, NLRC5 acts as an MHC class I specific trans activator by binding to and cooperating with the trimeric RFX transcription factor protein complex consisting of RFX5, RFXAP, and RFXANK/B (24). While the RFX complex is also essential for enhanced transactivation of MHC class II genes by CIITA, NLRC5 engages a unique S box sequence within the W/S motif thereby gaining specificity for a selected set of target genes (26). The MHC class I promoter also includes Enhancer A and an Interferon Stimulated Response Element (ISRE) to which transcription factors of the NFκB/Rel family and IRF1 bind, respectively (23, 27). Enhancer A and the ISRE element are important for both constitutive and cytokine-induced MHC class I expression (27, 28). In addition, both NLRC5 and CIITA are highly inducible by IFNγ stimulation. Thus, IFNɤ and other proinflammatory cytokines not only influence HLA expression directly through Enhancer A and ISRE elements but also act by upregulating NLRC5 and CIITA expression levels. Additional transcriptional control mechanisms beyond the immediate promoter region include long range enhancer-promotor interactions and the ability of CIITA and NLRC5 to influence chromatin opening through their interaction with chromatin modifiers, such as the histone acetyl transferases p300/CBP-binding protein (p300/CBP), general control of amino acid synthesis 5 (GCN5) and p300/CBP-associated factor (PCAF), thus enabling RNA polymerase II to initiate MHC gene transcription (29–31). These and other transcriptional regulators forming the MHC class I and II enhanceosome have been reviewed (25, 28, 32).

### Differences Between HLA-A, HLA-B, and HLA-C Promoter Structures

HLA-C has lower cell surface expression levels compared to HLA-A and HLA-B (33, 34). Furthermore, HLA-A and HLA-B have higher nucleotide diversity in the promoter region (1.8 and 1.9%, respectively) compared to the HLA-C promoter (0.9%), but no relationship has been found between promoter similarity and expression levels (35). Additional differences between the HLA-A, HLA-B and HLA-C promoter regions are found in Enhancer A. HLA-A contains two functional NFκB binding sites, κB1 and κB2. In the HLA-B and HLA-C promoters, κB2 contains major nucleotide variations that largely disable the NFκB binding. The transactivation of HLA-B by NFκB is facilitated by binding of transcription factor Specificity Protein 1 (SP1) to κB2. Interestingly, NFκB binding occurs neither at the κB1, nor at the κB2 site of the HLA-C Enhancer A (27, 36). Thus, direct NFκB transactivation seems to be restricted to the HLA-A and HLA-B genes. Using primary EVT and the EVT model cell line JEG3 we recently demonstrated that while IFNγ stimulation did indeed upregulate cell surface expression of HLA-C by an almost 5-fold change, IFNγ stimulation, expectedly, did not induce NFκB phosphorylation. In contrast, TNFα stimulation did induce NFκB phosphorylation in these cells and also increased HLA-C levels by 3-fold (16). These observations suggest that both, NFκB-dependent and NFκB-independent mechanisms may play a role in fine tuning the upregulation of HLA-C in trophoblasts upon cytokine stimulation.

Interestingly, a NK cell-specific HLA-C promoter was shown to produce a large array of differentially-spliced transcripts that vary in their ability to be translated into HLA-C protein and directly influenced the lytic activity NK acquire during their development (37). Here a polymorphism in the ETS/SP1-binding site in the HLA-C promoter was shown to influence HLA-C expression in NK cells, with individuals lacking an intact ETS-binding site having reduced HLA-C levels. Thus, the NK-intrinsic regulation of HLA-C, includes a distinct mechanism controlling its expression that influences NK development (37). Furthermore, identification of trophoblast-specific elements in the HLA-C core promoter further distinguished it from the HLA-A and HLA-B promoter (Figure 1) (38, 39). Here a specific transcriptional start site was observed in trophoblast cell lines ~30 bp upstream of the HLA-C transcriptional start site observed in other cell types. Enhanced HLA-C activity in trophoblast cell lines was mapped to the central enhanceosome region of the promoter, and mutational analysis identified changes in the ETS/RFX-binding region that generated a trophoblast-specific enhanceosome required for trophoblast-specific transcriptional activity (39). The mechanisms responsible for the specific expression of HLA-C in trophoblasts are likely to be distinct from HLA-G, which expression is controlled by a recently identified enhancer (enhancer L), 12 kb upstream of the HLA-G transcriptional start site and which is required for HLA-G expression in trophoblasts cell lines and primary EVT (39, 40). Here, the binding of CCAAT Enhancer Binding Protein Beta (C/EBPβ) and GATA Binding Protein 2/3 (GATA2/3) transcription factors and long-range chromatin looping were implicated in HLA-G enhancer L function. In conclusion these studies suggest that distinct mechanisms and unique cell type specific HLA-C transcription factors control HLA-C expression in somatic cell types, NK cells and trophoblasts.

### EVT Specific Factors Regulating HLA-C Expression in the Absence of HLA-A and HLA-B

While the transcriptional regulators that allow HLA-C, HLA-E, and HLA-G expression on EVT in the absence of HLA-A and HLA-B expression have not been identified thus far, a few studies have implicated other members of the NLR-family of proteins, to which both NLRC5 and CTIIA also belong, that can influence HLA-C expression by EVT. First, NLRP12 (formerly known as Monarch 1) was shown to regulate both the classical (HLA-A, HLA-B, and HLA-C) and non-classical (e.g., HLA-E and HLA-G) MHC class I protein and RNA expression at the promoter level (41). However, NLRP12 doesn't seem to localize to the nucleus and thus it is unclear how NLRP12 influences the promoter complex to regulate MHC transcription. Interestingly, NLRP12 is downregulated by TNFα and IFNγ and may only play a role in constitutive MHC class I expression. Of note, NLRP12 is predominantly expressed by dendritic cells, monocytes and granulocytes and its expression was also increased in EVT and JEG3 compared to decidual stromal cells (DSC) (16).

Secondly, NLRP2 which is highly expressed by EVT, was shown to suppress cell surface expression of HLA-C without affecting HLA-E and HLA-G expression on EVT (Figure 1D) (16). Furthermore, knock out of NLRP2 increased phosphorylation of NFκB in JEG3 and EVT upon TNFα stimulation, demonstrating the immune suppressive properties of NLRP2 in these cells. Interestingly, however was the observation that in the NLRP2 knock out JEG3 clones, HLA-C induction by TNFα and IFNγ was reduced suggesting that the presence of NLRP2 facilitates cytokine-induced HLA-C expression. By fine tuning HLA-C expression levels on EVT, NLRP2 may contribute to shape immune cell responses to EVT and balance both immune tolerance and immunity (16). These observations also support the notion that NLRP12 and NLRP2 and possibly other proteins of the NLR family have additional functions beyond pattern recognition and induction of proinflammatory responses that includes the control of MHC expression (42).

### HLA-C Cell Surface Expression Varies Widely Across Individuals in an Allele-Specific Manner

Variations in HLA-C cell surface expression levels have been shown to influence the efficacy of cellular immune responses to viral-, allo- and self-antigens (10, 11, 22). High HLA-C protein expression on the cell surface was associated with protection against the HIV-1 virus, increased cytotoxic T lymphocyte (CTL) responses and increased frequency of HIV escape mutations, suggesting that high HLA-C expression exerts a selection pressure on the virus (10). High HLA-C expression levels also correlate with increased risk of Crohn's disease (11), and in cases of unrelated haematopoietic transplantation, with poor outcome and graft-vs.-host disease (22). Particularly the observed differences in HLA-C restricted allo-responses and HLA-C restricted anti-viral responses may have clinical implications for pregnancy outcome, but the correlation between HLA-C expression levels and pregnancy outcome has not been investigated thus far.

Variation in HLA-C expression between different HLA-C alleles have been attributed to an atypical dimorphic binding site for OCT1 (also known as POU2F1), located ~800 bp upstream of the HLA-C transcriptional start site. OCT1 is a member of the POU transcription factor family with unusual importance in embryogenesis (43). The dimorphism results from a single SNP (rs2395471) which seems to affect the affinity of OCT1 for the site and hence HLA-C promoter activity and HLA-C protein levels (33, 44). OCT1 was shown to bind with lower affinity to the G than to the A allele, resulting in lower HLA-C promoter activity and lower HLA-C cell surface levels. This polymorphism is responsible for up to ~36% of the difference in HLA-C cell surface levels. These observations did not address, however, whether OCT1 is essential for HLA-C expression or may only serve as factor that fine-tunes the level of HLA-C transcription.

Another sequence polymorphism in the 3′ untranslated region (UTR) of HLA-C, involves the binding site for miR-148a. HLA-C alleles that have an intact miR-148a binding site (e.g., C^*^07 and C^*^03), have low protein expression due to miRNA mediated inhibition (Figure 1B). In contrast, about seven HLA-C alleles (including HLA-C^*^05 and HLA-C^*^08) escape miR-148a binding due to a deletion in the miR binding site, and these proteins are expressed at higher levels. This miR-148a binding variation is unique for HLA-C, as all HLA-A molecules have an intact binding site for miR148a and all HLA-B molecules escape the miR148a binding (11, 45, 46). Other variations in HLA-C cell surface expression did not correlate with mRNA expression levels and were attributed to post-transcriptional and structural diversity of the HLA-C proteins (34). Most importantly, differences in the peptide binding groove and the diversity of peptides bound by different HLA-C alleles (Figure 1C) affected protein stability and thus impacted HLA-C cell surface expression levels (34).

## Recognition of Fetal HLA-C Allo-Antigens by Maternal NK and T Cells

While the expression of a polymorphic paternally inherited HLA-C antigen by EVT provides a target for maternal NK cells and T cells to recognize and respond to, the HLA-C cell surface expression levels influence how this response is shaped. As HLA-C^+^ HLA-G^+^ EVT invade maternal uterine tissues they encounter maternal leukocytes of which ~70–80% are decidual NK cells (dNK), 5–15% are decidual T cells, and ~10–15% are decidual macrophages (dMΦ) in first trimester pregnancy. These proportions change dramatically during the course of pregnancy so that at term pregnancy, T cells are the predominant decidual lymphocyte population comprising 40–70% of CD45^+^ leucocytes, but dNK and dMΦ remain present in relatively high proportions of ~20–50 and ~10–15%, respectively (47–49).

### HLA-C Recognition by Decidual NK Cells (dNK)

NK cells specifically recognize two groups of HLA-C allotypes, HLA-C1, and HLA-C2 group alleles, based on natural amino acid substitutions at position 80 of the HLA-C heavy chain, here HLA-C1 has asparagine and HLA-C2 group molecules have a lysine (50). NK cells were shown to carry killer cell Ig-like receptors (KIRs) with discrete specificity for HLA including HLA-C1, using KIR2DL2 and KIR2DL3 and HLA-C2 group allotypes using KIR2DL1 and KIR2DS1. Other KIRs were shown to recognize some HLA-A and HLA-B allotypes (7, 51). Due to rearrangements in the KIR gene cluster, which included duplications and deletions, everyone possesses a different combination and different number of inhibitory and activating KIR genes (52, 53). Additionally, within each individual, the process of how NK cells maturate strongly depends on whether within this individual HLA allotypes for the KIR are present or not. Furthermore, the HLA-C protein expression levels directly influenced the lytic activity NK acquire during their development (37). Thus, during pregnancy maternal KIR haplotypes, NK cell education as well as the maternal and fetal HLA-C allotype combinations, differ in each pregnancy and shape the interactions of dNK with invading EVT.

**Figure 2:**
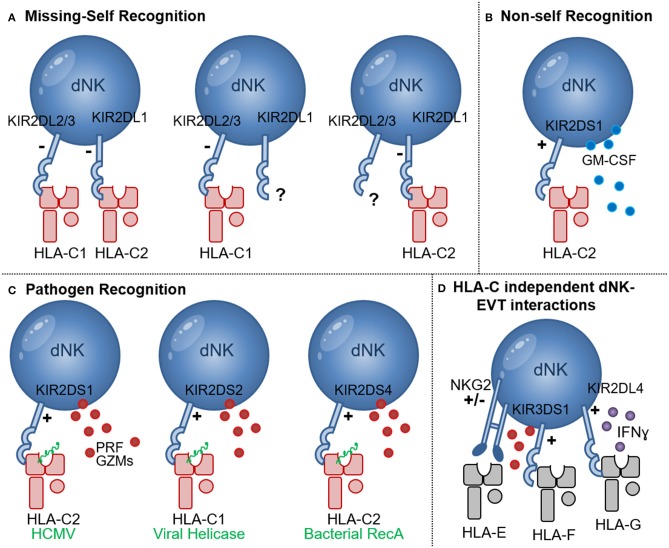
NK cell recognition of HLA-C. **(A)** Missing-self recognition leads to NK activation when the HLA-C group ligand for a KIR is absent (e.g., when HLA-C2 is absent in the presence of KIR2DL1 or HLA-C1 is absent in the presence of KIR2DL2/3); **(B)** Recognition of allogeneic HLA-C2 molecules (red) may occur through binding of KIR2DS1 to HLA-C2 molecules. Upon HLA-C2—KIR2DS1 interaction GM-CSF secretion by dNK has been shown; **(C)** Pathogen derived peptides (green) presented by HLA-C1 and HLA-C2 molecules can activate NK cells expressing the activating receptors KIR2DS1, KIR2DS2, and KIR2DS4 in processes that may enhance NK cytotoxicity, release of perforin (PRF) and granzymes (GZMs) and pathogen clearance; **(D)** HLA-C independent NK-EVT interactions include HLA-E and NKG2A/C as well as HLA-F and KIR3DS1 interactions that may lead to degranulation and release of perforin (PRF) and granzymes (GZMs). Interaction of HLA-G and KIR2DL4 was shown to inhibit dNK cytotoxicity and promote IFNɤ secretion.

dNK are specifically shown to have a KIR expression profile that is skewed in their high ability to recognize the HLA-C allotypes HLA-C1, utilizing the inhibitory KIR2DL2/3 receptors, and HLA-C2 using inhibitory KIR2DL1 and the activating KIR2DS1 receptors (Figure 2) (54). In 2004 Hiby et al., demonstrated that combinations of maternal KIR and fetal HLA-C genes influence the risk of preeclampsia and reproductive success (55). In this study it was shown that mothers lacking most or all activating KIR, named the KIR-AA genotype, in combination with a fetus expressing HLA-C belonging to the HLA-C2 group, were at an increased risk of preeclampsia. Later it was shown that responses generated by the activating KIR2DS1 receptor binding to HLA-C2 resulted in secretion of cytokines, such as Granulocyte-Macrophage Colony-Stimulating Factor (GM-CSF), which enhances migration of primary trophoblasts *in vitro* (56). However, the association between KIR-AA genotype and HLA-C2 and the increased risk of pregnancy complications has not been consistently reported (57, 58). Furthermore, another study didn't confirm the secretion of GM-CSF by KIR2DS1^+^ dNK during *in vitro* co-culture with HLA-C2^+^ EVT (59). KIR2DS1^+^ dNK acquired more HLA-G, compared to KIR2DS1- dNK, during co-culture with primary EVT in a process called trogocytosis (60). dNK acquired HLA-G from EVT through direct cell-cell contact in which actin-ring formations, typical of an immune synapse, were formed between dNK and EVT. This however didn't result in EVT lysis by dNK. Additional genetic studies have further demonstrated that the presence of KIR2DS5 was associated with lower risk of developing pregnancy complications in African women, and KIR2DS5 genotypes that recognize HLA-C2 allotypes are common among Africans and absent from Europeans (61). In contrast, the protective effect of KIR2DS1 seems to be characteristic of European populations (61, 62). The presence of activating KIR was also associated with an increased birth weight (63). Although all studies described here point toward an increased interaction of KIR2DS1^+^ dNK with HLA-C2^+^ EVT, more detail on the mechanism underlying the protective effects of KIR2DS1 in pregnancy is required. Other lines of investigation should also include the possibility that HLA-C allo-recognition by dNK contributes to limiting EVT invasion and preventing deep invasion and placentation that is associated with placenta accreta, increta, and percreta, conditions that involve abnormal adherence of the placental trophoblasts to the uterine myometrium which can lead to fatal bleeding if not clinically managed (64).

### HLA-C Specific CD8^+^ T Cell Responses

**Figure 3:**
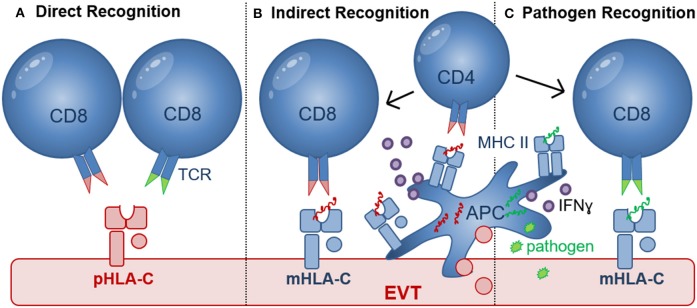
Recognition of HLA-C by CD8^+^ T cells. **(A)** Direct recognition of allogeneic paternal HLA-C (pHLA-C, depicted in red) occurs through allo-specific CD8 T cells (left) and cross-reactive memory CD8^+^ T cells (right); **(B)** Indirect recognition of allo-antigens occurs when APC process and present allogeneic peptide antigens to activate CD8^+^ T cells in a process where CD4^+^ T cells may be involved. Differentiation of CD4^+^ and CD8^+^ T cells depends on the presence of pro-inflammatory (e.g., IFNɤ, TNFα, and IL-12) and anti-inflammatory (e.g., IL-10) cytokines produced by APC or other cell types in the placental microenvironment. Activated HLA-C restricted CD8^+^ T cells may recognize paternal peptide antigens presented in by maternal HLA-C (mHLA-C, depicted in blue); **(C)** Pathogen-derived peptide antigens (green) presented by HLA-C can activate HLA-C-restricted pathogen-specific CD8^+^ T cells. Efficient priming of pathogen-specific CD8^+^ T cells depends on antigen presentation by APC, CD4^+^ T cell help as well as the presence of pro-inflammatory (e.g., IFNɤ, TNFα, and IL-12) and anti-inflammatory (e.g., IL-10) cytokines.

Maternal decidual CD8^+^ T cells are key cells that can directly recognize allogenic HLA-C molecules of paternal origin during pregnancy (Figure 3) (65). Recognition of allogeneic HLA molecules largely depends on, (i) the differences in amino acid motifs (between donor/recipient) in the α1 and α2 domains of the HLA molecule which are relevant for HLA-TCR binding, (66, 67); (ii) the selection of peptides presented by the foreign MHC molecules (68); (iii) the TCR repertoire of the responder T cell pool; and (iv) the HLA cell surface expression levels on the target cells (9, 22). Previously, HLA-C has been shown to elicit a direct cytotoxic response by CD8^+^ T cells during allogeneic organ and hematopoietic stem cell (HSC) transplantation (66, 69). However, the percentage of donor/patient pairs with a detectable CTL response was lower in the HLA-C mismatched group (~38%) compared to donor/patient pairs with a single HLA-A or HLA-B mismatch (~65%) (66, 69). Furthermore, the HLA-C cell surface expression level significantly influenced CTL reactivity, with the donor cells expressing the highest HLA-C levels eliciting stronger CTL responses (9, 22). The expression level of the patient's mismatched HLA-C allotype was also linked to transplant outcome (9, 22). The importance of recognition of fetal HLA-C in pregnancy was further demonstrated in a recent study suggesting that HLA-C antibodies may contribute to the ethology of miscarriage (70). Furthermore, fetus-specific CD8^+^ CTL responses to minor Histocompatibility antigens (mHags) were initiated in maternal peripheral blood during uncomplicated pregnancies (71–73). Here mHAg-specific responses had HLA-A and HLA-B restriction, but HLA-C-restricted mHAg-specific responses have not been investigated thus far. Combined, these studies demonstrate that CTL responses to allogeneic HLA-C and mHAg are generated and can be detected in peripheral blood in high proportions of pregnant woman. However, during pregnancy a maternal-fetal HLA-C mismatch is associated with immune tolerance possibly due to the tolerogenic microenvironment at the maternal-fetal interface (74, 75).

Maternal decidual CD8^+^ T cells form a minority of leukocytes present in first trimester decidual tissue (~2–7% of CD45^+^ cells) but their proportion increases up to ~30% in term pregnancy decidua. Decidual CD8^+^ T cells mainly consist of highly differentiated CD8^+^ effector-memory T cells, suggesting that antigens are present at the maternal-fetal interface that can attract antigen-specific CD8^+^ T cells responses (76). Decidual CD8^+^ T cells were shown to have high expression of the co-inhibitory molecules Programmed Cell Death 1 (PD1), Cytotoxic T-Lymphocyte Associated Protein-4 (CTLA4), and Lymphocyte Activation Gene-3 (LAG3) and low expression of cytolytic molecules suggesting that the decidual microenvironment reduces CD8^+^ effector T cell function to maintain placental tolerance (76, 77). However, upon stimulation *in vitro* decidual CD8^+^ T cells degranulated, proliferated, produced IFNγ, TNFα, perforin, and granzyme B, demonstrating that decidual CD8^+^ T cells are not permanently suppressed and retain the capacity to respond to proinflammatory events, such as infections (77). The balance between transient dysfunction of decidual CD8^+^ T cells that are permissive of placental and fetal development, and reversal of this dysfunctional state, is crucial for placental tolerance and immunity to placental infections. In mice activation of T cells with direct specificity for paternal allogeneic MHC expressed by mouse trophoblast cells was observed in one study (78), but activation of allo-reactive T cells was not detected in another (79). Thus, both human and murine studies demonstrate that some but not all maternal-fetal MHC mismatches induce T cell responses. More importantly, these studies reveal that the presence of maternal CD8^+^ T cells with a direct specificity for fetal MHC was not associated with pregnancy complications or pregnancy failure.

### HLA-C Recognition and Establishment of T Cell Tolerance

**Figure 4:**
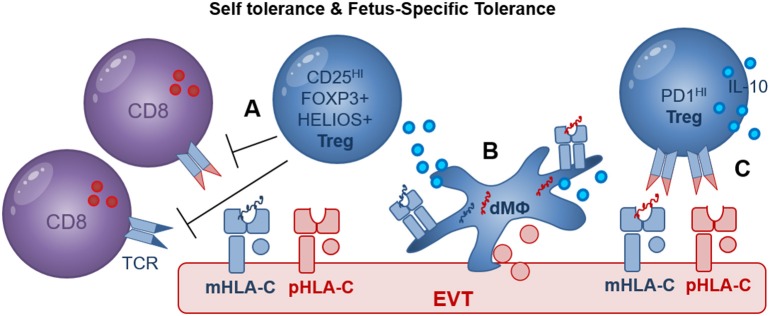
Treg mediated recognition of HLA-C may include self- and fetus-specific tolerance. **(A)** CD25^HI^ FOXP3^+^ Treg were shown to suppress fetus-specific and non-specific lymphocyte responses; maternal HLA-C (mHLA-C, blue), paternal HLA-C (pHLA-C, red); **(B)** Decidual macrophages (dMΦ) and EVT directly increase the proportion of FOXP3^+^ Treg during *in vitro* co-culture assays; maternal peptides (blue), paternal peptides (red); **(C)** EVT directly increase the proportion of PD1^HI^ Treg in a process where HLA-C and TCR interaction may play a role. In addition, efficient induction of FOXP3^+^ Treg and PD1^HI^ Treg by dMΦ and EVT may depend on cell surface receptors as well as secreted cytokines including IL-10.

Maternal regulatory T cells (Treg) are of unique importance in establishing immune tolerance to invading EVT and preventing detrimental inflammatory responses to fetal and placental antigens. Adoptive transfer of CD4^+^CD25^+^ Treg depleted lymphocytes to pregnant mice resulted in an increased resorption rate in allogeneic but not in syngeneic matings and CD3^+^ T cells were observed in placental tissues (80). Similarly, in another murine study mitigation of FOXP3^+^ Treg induction during pregnancy was used and resulted in antigen-specific fetal loss (81) and adoptive transfer of Treg from normal pregnant mice to abortion prone mice prevented fetal resorption in the abortion prone mouse model (82). Thus, these studies demonstrate that Treg play a key role in preventing rejection of allogeneic fetuses and maternal allo-specific lymphocytes can mediate fetal rejection in the absence of Treg. CD4^+^CD25^HI^FOXP3^+^ Tregs are found at high levels in human decidual tissues in first trimester pregnancy and term pregnancy decidua basalis and decidua parietalis tissues (83–85). Decidual Treg and were shown to suppress fetus-specific and non-specific responses (Figure 4) (47). Most interestingly, HLA-C mismatched pregnancies had increased levels of CD4^+^CD25^dim^ activated T cells and increased levels of functional decidual CD4^+^CD25^HI^ Tregs, compared to HLA-C matched pregnancies (74). This suggests that maternal T cells may specifically recognize fetal HLA-C, and that this activation promotes Treg differentiation. In this study it was suggested that indirect presentation of HLA-C antigen by APC may be responsible for the increase in CD4^+^(83) CD25^HI^ Tregs. But recent studies demonstrate that *in vitro* co-culture of naive CD4^+^ T cells with EVT, directly increased the proportion of CD4^+^FOXP3^+^ Tregs, compared to CD4^+^ T cells cultured alone (59, 86, 87). Moreover, HLA-G^+^ EVT, but not dMΦ, also increased the proportion of PD1^HI^ Treg, in a process that may depend on HLA-C and TCR interactions (88). Here the PD1^HI^IL-10^+^ Treg suppressed CD4^+^ T cell proliferation and also increased IL-10 expression by CD4^+^ and CD8^+^ T cells possibly resulting in a positive feedback loop sustaining T cell suppression while inducing additional IL-10 secreting Tregs (88).

The question of whether decidual Treg are natural Treg (nTreg) generated in the thymus with specificity for self-antigens or induced Treg (iTreg) generated in the periphery with specificity for paternal antigens remains to be answered (89, 90). Co-culture of CD4^+^ T cells with EVT or dMΦ both significantly increased the expression of FOXP3 and HELIOS, advocating for either a local expansion of FOXP3^+^ and HELIOS^+^ nTreg or a possible *de novo* induction of FOXP3^+^ and HELIOS^+^ iTreg (88). Clonally expanded CD4^+^CD25^HI^CD127^−^CD45RA^−^ Treg populations were also observed in term pregnancy decidua (91) but this study did not investigate the specificity of Treg suppression for self- or fetal-antigens. Evidence supporting the clinical relevance of Treg in pregnancy includes studies demonstrating decreased proportions of decidual FOXP3^+^ and HELIOS^+^ Tregs in cases of unexplained miscarriage compared to miscarriage with karyotype abnormalities (92, 93) as well as in preeclampsia compared to healthy control pregnancies (94–96). Further investigation of the multiple decidual Treg populations by studying their presence, functionality and specificity for HLA-C should reveal their role in fetus specific immune tolerance and in development of pregnancy complications.

## Pathogen Recognition in the Context of HLA-C

The maternal immune system must establish tolerance for fetal and placental antigens and provide protective immunity to fight infections (65). While NK cells utilize KIR and HLA-C interactions to clear pathogen infected target cells, when EVT are infected, maternal HLA-C is also the only molecule that can present pathogen-derived peptides to antigen-specific memory CD8^+^ T cells and provide adaptive immunity (65, 97). Although pregnant women can generate protective immune responses to a variety of pathogens, when infections occur during pregnancy, they can cause severe maternal and fetal morbidity (98–101). Human placenta has no microbiome, but many pathogens infect the placenta before transmission to the fetus occurs (102, 103). Pathogens that can directly infect trophoblasts and other placental cells, include but are not limited to: Human Cytomegalovirus (HCMV), Hepatitis C virus (HCV), Herpes Simplex virus (HSV), Human Papillomavirus (HPV), Zika Virus (ZIKV), Rubella Virus, Varicella Zoster Virus (VZV), parvovirus B19, Listeria *Monocytogenes* (*L. monocytogenes*), Toxoplasma g*ondii*, Ureaplasma *urealyticum*, Mycoplasma *hominis* (100, 104–111). Besides the severe congenital disease that occurs when pathogens transmit from mother to fetus, infections during pregnancy are also associated with recurrent spontaneous abortions (RSA), preterm birth, intrauterine growth restriction and preeclampsia (112–116). Very limited experimental data is present on how maternal immune cells respond to pathogens and provide immunity at the maternal-fetal interface.

### HLA-C and KIR Interactions Enhances dNK Responses to Infection

In addition to the protective effects of HLA-C and activating KIR interactions during pregnancy complications [discussed in section HLA-C Recognition by Decidual NK Cells (dNK)], individuals who carry activating KIR also have a significantly improved outcome during viral infections including HCMV, HIV, HCV, and HPV infections (117–121). dNK (but not pNK), expressing KIR2DS1 had an increased ability to degranulate in response to HCMV-infected HLA-C2^+^ DSC during *in vitro* co-cultures (122). Engagement of KIR2DS1 with HLA-C2 molecules was shown to be dependent on peptides presented by HLA-C2 (123). Importantly, modulation of HLA-C2 by HCMV peptides stimulated KIR2DS1 recognition by NK cells, providing a molecular basis for the increased degranulation response of KIR2DS1^+^ dNK to HCMV infection (Figure 2C) (124). Newer studies investigating the ligands binding to other activating KIR demonstrated roles for HLA-C in presenting bacterial and viral peptides to increase KIR binding and NK cell cytotoxicity. The activating receptor KIR2DS4 detects a highly conserved peptide sequence motif derived from bacterial recombinase A (RecA) when it's presented by HLA-C^*^05:01, an HLA-C2 group molecule (Figure 2C) (125). This study predicts that over 1,000 bacterial species, including *Helicobacter, Chlamydia, Brucella*, and *Campylobacter* species, could activate NK cells through KIR2DS4. Thus, human NK cells also contribute to immune defense against bacteria through recognition of a conserved RecA epitope presented by HLA-C^*^05:01 (125). In addition, KIR2DS2 recognized conserved peptide epitopes of viral helicases, in the context of HLA-C^*^01:02, an HLA-C1 group molecule (Figure 2C) (126). Viral helicases from hepatitis C virus and a number of flaviviruses including Dengue, Zika, and Yellow fever viruses presented in the context of HLA-C^*^0102 to KIR2DS2^+^ NK cells, was sufficient to inhibit HCV and Dengue virus replication. The study illustrates that a KIR receptors have evolved to activate NK cells in response to multiple pathogenic viruses (126). Targeting short, but highly conserved, viral and bacterial peptides provide non-rearranging immune receptors with an efficient mechanism to specifically recognize multiple, highly variable, pathogens, a feature that is generally associated with rearranging T cell and B cell receptors. Interestingly however a combination of the activating receptor KIR2DS3 in combination with a single nucleotide polymorphism of the IL28B gene, significantly increased the risk of chronic disease in hepatitis C virus infection, possibly through a IL28 mediated inhibition of IFNɤ by NK cells (127). Other studies also demonstrated that diminished inhibitory responses through KIR2D and HLA-C interactions, confers protection against HCV (128–130) and influences the development of severe influenza (131). Many HLA-C independent interactions between NK cells and EVT may contribute to inhibition and activation of dNK cytotoxicity, e.g., HLA-G and KIR2DL4 interaction favors IFN production but inhibits NK cytotoxicity (Figure 2D) (132), NKG2 receptors mediate responses through HLA-E as well as cellular stress ligands (133). Open conformers of HLA-F, which are HLA-F heavy chains devoid of peptide and/or β2-microglobulin (β2m), are high-affinity ligands of the activating NK-cell receptor KIR3DS1 and may play a role in limiting HIV-1 infection (119, 134). Furthermore, a recent report identified a unique subset of dNK expressing high levels of the activating HLA-E receptor NKG2C and the HLA-G receptor LILRB1 in multigravid woman compared to woman with a first pregnancies. These dNK secreted high levels of IFN-γ and VEGFα and may play a role in enhanced placentation in repeated pregnancies (135).

The mechanisms by which activating KIR and HLA-C interactions reduce the risk of pregnancy complications and enhance immunity to infections during pregnancy should be investigated in more molecular detail. Enhanced systemic NK mediated immunity to infection likely reduces systemic pathogen burden and can limit pathogens from infecting placental tissues. The increased capacity of KIR2DS1 to clear HCMV-infected DSC in the placenta suggests that activating KIR also enhance dNK mediated placental immunity (122). However, the failure of dNK to kill EVT, even when infected with HCMV, may reduce the risk of immune rejection of EVT and placental tissues but promote the spread of infection and contribute to virus-induced placental pathology and development of complications later in pregnancy (97). These mechanisms may be related to how KIR2DS1, expressed by dNK, reduces development of severe pregnancy complications, such as miscarriages and preterm delivery. Increasing pNK and dNK responses by expansion of KIR expressing NK cells or utilizing chimeric antigen receptor NK cells are currently being investigated for treatment of cancer and chronic infections (136, 137). Utilizing these approaches to enhance HLA-C and KIR interactions in pNK and dNK has therapeutic potential to limit placental infection as well as mother to fetal transmission of a variety of pathogens. A focus on controlling infections during pregnancy may also help limit infection induced placental pathology and complications of pregnancy, such as miscarriage and spontaneous preterm birth that are related to infection (115, 138).

### HLA-C-Restricted Pathogen-Specific CD8^+^ T Cell Responses

The failure of dNK to respond to HCMV-infected EVT during *in vitro* co-culture (122), may leave decidual CD8^+^ T cells as the predominant effector cell to clear pathogen-infected EVT. HCMV sero-positivity was shown to dramatically alter the maternal CD8^+^ T cell repertoire during pregnancy (139) and similar to HIV, T cell responses to HCMV rely heavily on HLA-C-restricted responses because both viruses downregulate HLA-A and HLA-B upon infection (10, 140). During HIV and HCMV infection, HLA-C-restricted CD8^+^ CTL responses were shown to comprise as much as 54% of the total response in peripheral blood and were functionally and phenotypically identical to HLA-A- and HLA-B-restricted CTL (140, 141). Seropositive women during late pregnancy demonstrated an accumulation of highly differentiated HCMV-specific T-cells (139). Moreover, increased percentages of HCMV and EBV-specific CD8^+^ T cells were also found in decidual tissue compared with peripheral blood after uncomplicated pregnancy (142). These decidual virus-specific CD8^+^ memory T cells were able to produce IFNγ and were restricted to recognize viral peptides presented by HLA-A or HLA-B molecules. Thus, these decidual CD8^+^ T cells may provide cellular immunity for infected maternal cells that express HLA-A and HLA-B and limit the spread of infection to trophoblasts and/or the fetus. However, no data are currently available as to whether HLA-C-restricted virus-specific CTL are present and can provide immunity when EVT are infected (Figure 3C).

### Virus Specific CTL Cross-React With HLA-C Allo-Antigens

A substantial proportion of virus-specific CD4^+^ and CD8^+^ memory T cells [for e.g., HCMV, Epstein–Barr virus (EBV), (VZV), and Influenza Virus] were shown to cross-react with non-self allogeneic HLA molecules, including HLA-A, HLA-B, and HLA-DR molecules. In this case, the allogeneic HLA reactivity and virus specificity were mediated via the same TCR (143, 144). More recently, cross reactivity of a HLA-B^*^08:01-restricted EBV-specific peripheral blood T cell clone, showed significant alloreactivity against HLA-C^*^01:02 and in the same study cross reactivity of HLA-C^*^06:02-restricted HCMV-specific peripheral blood CD8^+^ T cell line against HLA-C^*^03:02 allele was detected (145). Although decidual CD8^+^ T cells contain higher proportions of virus specific CTL, these have not been investigated for cross reactivity (70) and no cross reactivity against HLA-E and -G molecules was detected (145). This demonstrates that cross reactivity of virus-specific CD8^+^ T cells against HLA-C can occur (Figure 3A), but cross reactivity to HLA-C allotypes was found to be about 10-fold lower than to HLA-A, HLA-B, and HLA-DR allotypes. Most importantly, cross reactivity has been shown to depend on both the peptides presented by allogeneic HLA molecules and the tissue and cell types used. EVT have been shown to express many ligands including Programmed Cell Death Ligand-1 (PDL1), Cytotoxic and Regulatory T Cell Molecule (CRTAM), B7H3, Polio Virus Receptor (PVR), and secrete inhibitory cytokines that may inhibit direct CD8 cytotoxicity (59). Furthermore, co-culture of blood CD8^+^ T cells or decidual CD8^+^ T cells with allogeneic EVT did not result in degranulation (59, 77). Further testing of cross reactive CD8^+^ T cells against HLA-C allotypes expressed on EVT in healthy pregnancy and pregnancy pathology will determine their role in development of pregnancy complications.

### Viral Infections Influence Treg Stability and Enhance CTL Reactivity

Uncontrolled placental viral (and bacterial) infections also provide a pro-inflammatory milieu that can alter the stability and function of Treg and enhance alloreactivity (65, 146). In transplant recipients, infections have been associated with failure to induce transplant tolerance and allograft rejection even after long periods of transplant tolerance (146, 147). HCMV infection of EVT did not diminish the ability of EVT to increase FOXP3^+^ and PD1^HI^ Tregs (88), suggesting that HCMV infection does not alter the capacity of EVT to promote immune tolerance. This finding is in line with the observation that dNK fail to degranulate in response to HCMV-infected EVT and thus also maintain immune tolerance in the presence of infection (122). Future studies need to investigate the impact of other placental cell types (e.g., dMΦ and DSC) and changes in inflammatory factors of the placental microenvironment during infection on decidual Treg stability and Treg induction in the presence of infection.

## Outstanding Questions

- What are the regulatory mechanisms by which EVT express HLA-C, HLA-E, and HLA-G in the absence of HLA-A and HLA-B?
- Do high HLA-C cell surface expression levels enhance immunity to placental infection and/or generation of HLA-C reactive decidual T cell responses?
- Are decidual CD8^+^ T cells able to recognize and respond to viral, fetal and/or placental antigens in the context of HLA-C expressed by EVT?
- Do maternal regulatory T cells provide specific immune tolerance to fetal HLA-C mismatches in the placenta?
- What are the molecular and cellular mechanisms by which activating KIR and HLA-C interactions reduce the risk of pregnancy complications and enhance immunity to infections?

## Concluding Remarks

The co-expression of maternally and paternally inherited HLA-C by EVT provides both a self and a non-self ligand for maternal decidual CD4^+^ Treg, CD8^+^ Teff, and dNK to establish self- and fetus-specific immune tolerance. The maternally inherited HLA-C is the only molecule that can present pathogen-derived peptides to antigen-specific memory CD8^+^ T cells to provide adaptive immunity when EVT become infected. However, the expression of paternally inherited polymorphic HLA-C by EVT also provides a potential ligand for cytolytic CD8^+^ Teff and dNK, possibly resulting in detrimental inflammatory responses that are associated with pregnancy complications. Thus, HLA-C expression by EVT has a unique and dual role in maternal-fetal immune tolerance and immunity to placental infections (65). High HLA-C expression levels on the cell surface may enhance detrimental inflammatory and CTL responses to maternal-fetal HLA-C mismatches in pregnancy as was shown in transplant patients (9, 22). However, high HLA-C levels in pregnancy can also be beneficial and contribute to immune protection to a wide variety of infections and diminish infection related pregnancy complications (10). Further investigations, as to how EVT express HLA-C in the absence of HLA-A and HLA-B, as well as the role of individual HLA-C expression levels in shaping the maternal dNK, Teff and Treg responses to HLA-C will be key in understanding the development of pregnancy complications and preventing maternal to fetus transmission of infections.

## Author Contributions

HP and TT: writing—original draft. TM, QL, JS, and TT: writing—review and editing. TT: visualization.

### Conflict of Interest

The authors declare that the research was conducted in the absence of any commercial or financial relationships that could be construed as a potential conflict of interest.

## References

[1] Thorsby E Sandberg L Lindholm A Kissmeyer-Nielsen F. The HL-A system: evidence of a third sub-locus. Scand J Haematol. (1970) 7:195–200. 10.1111/j.1600-0609.1970.tb01887.x 5447964

[2] Carter AM. Comparative studies of placentation and immunology in non-human primates suggest a scenario for the evolution of deep trophoblast invasion and an explanation for human pregnancy disorders. Reproduction. (2011) 141:391–6. 10.1530/REP-10-0530 21273370

[3] Snary D Barnstable CJ Bodmer WF Crumpton MJ. Molecular structure of human histocompatibility antigens: the HLA-C series. Eur J Immunol. (1977) 7:580–5. 10.1002/eji.1830070816 332508

[4] Zemmour J Parham P Distinctive polymorphism at the HLA-C locus: implications for the expression of HLA-C. J Exp Med. (1992) 176:937–50. 10.1084/jem.176.4.937 1383381 PMC2119399

[5] Robinson J Waller MJ Parham P de GN Bontrop R Kennedy LJ. IMGT/HLA and IMGT/MHC: sequence databases for the study of the major histocompatibility complex. Nucleic Acids Res. (2003) 31:311–4. 10.1093/nar/gkg070 12520010 PMC165517

[6] Ljunggren HG Karre K. In search of the ‘missing self': MHC molecules and NK cell recognition. Immunol Today. (1990) 11:237–44. 10.1016/0167-5699(90)90097-S 2201309

[7] Parham P. The genetic and evolutionary balances in human NK cell receptor diversity. Semin Immunol. (2008) 20:311–6. 10.1016/j.smim.2008.10.002 19036608 PMC3205964

[8] McIntyre JA Faulk WP. Recurrent spontaneous abortion in human pregnancy: results of immunogenetical, cellular, and humoral studies. Am J Reprod Immunol. (1983) 4:165–70. 10.1111/j.1600-0897.1983.tb00272.x 6234813

[9] Israeli M Roelen DL Carrington M Petersdorf EW Claas FH Haasnoot GW. Association between CTL precursor frequency to HLA-C mismatches and HLA-C antigen cell surface expression. Front Immunol. (2014) 5:547. 10.3389/fimmu.2014.00547 25386183 PMC4209872

[10] Apps R Qi Y Carlson JM Chen H Gao X Thomas R. Influence of HLA-C expression level on HIV control. Science. (2013) 340:87–91. 10.1126/science.1232685 23559252 PMC3784322

[11] Kulkarni S Qi Y O'HUigin C Pereyra F Ramsuran V McLaren P Genetic interplay between HLA-C and MIR148A in HIV control and Crohn disease. Proc Natl Acad Sci USA. (2013) 110:20705–10. 10.1073/pnas.1312237110 24248364 PMC3870724

[12] Ellis SA Sargent IL Redman CW McMichael AJ. Evidence for a novel HLA antigen found on human extravillous trophoblast and a choriocarcinoma cell line. Immunology. (1986) 59:595–601. 3804380 PMC1453327

[13] Kovats S Main EK Librach C Stubblebine M Fisher SJ DeMars R. A class I antigen, HLA-G, expressed in human trophoblasts. Science. (1990) 248:220–3. 10.1126/science.2326636 2326636

[14] Apps R Murphy SP Fernando R Gardner L Ahad T Moffett A. Human leucocyte antigen (HLA) expression of primary trophoblast cells and placental cell lines, determined using single antigen beads to characterize allotype specificities of anti-HLA antibodies. Immunology. (2009) 127:26–39. 10.1111/j.1365-2567.2008.03019.x 19368562 PMC2678179

[15] Ferreira LM Meissner TB Tilburgs T Strominger JL. HLA-G: at the interface of maternal-fetal tolerance. Trends Immunol. (2017) 38:272–86. 10.1016/j.it.2017.01.009 28279591

[16] Tilburgs T Meissner TB Ferreira LMR Mulder A Musunuru K Ye J NLRP2 is a suppressor of NF-kB signaling and HLA-C expression in human trophoblasts dagger, double dagger. Biol Reprod. (2017) 96:831–42. 10.1093/biolre/iox009 28340094 PMC5803763

[17] van den Elsen PJ Gobin SJ van der SN Datema G Vietor HE van der Stoep N. Transcriptional control of MHC genes in fetal trophoblast cells. J Reprod Immunol. (2001) 52:129–45. 10.1016/S0165-0378(01)00115-2 11600183

[18] Meissner TB Li A Biswas A Lee K-H Liu Y-J Bayir E. NLR family member NLRC5 is a transcriptional regulator of MHC class I genes. Proc Natl Acad Sci USA. (2010) 107:13794–9. 10.1073/pnas.1008684107 20639463 PMC2922274

[19] Neerincx A Rodriguez GM Steimle V Kufer TA. NLRC5 controls basal MHC class I gene expression in an MHC enhanceosome-dependent manner. J Immunol. (2012) 188:4940–50. 10.4049/jimmunol.1103136 22490867

[20] Staehli F Ludigs K Heinz LX Seguin-Estevez Q Ferrero I Braun M. NLRC5 deficiency selectively impairs MHC class I- dependent lymphocyte killing by cytotoxic T cells. J Immunol. (2012) 188:3820–8. 10.4049/jimmunol.1102671 22412192

[21] Steimle V Otten LA Zufferey M Mach B. Complementation cloning of an MHC class II transactivator mutated in hereditary MHC class II deficiency (or bare lymphocyte syndrome). Cell. (1993) 75:135–46. 10.1016/S0092-8674(05)80090-X 8402893

[22] Petersdorf EW Gooley TA Malkki M Bacigalupo AP Cesbron A Du Toit E, International Histocompatibility Working Group in Hematopoietic Cell T. HLA-C expression levels define permissible mismatches in hematopoietic cell transplantation. Blood. (2014) 124:3996–4003. 10.1182/blood-2014-09-599969 25323824 PMC4271183

[23] van den Elsen PJ Peijnenburg A van Eggermond MC Gobin SJ. Shared regulatory elements in the promoters of MHC class I and class II genes. Immunol Today. (1998) 19:308–12. 10.1016/S0167-5699(98)01287-0 9666603

[24] Meissner TB Liu Y-J Lee K-H Li A Biswas A van Eggermond MCJA. NLRC5 cooperates with the RFX transcription factor complex to induce MHC class I gene expression. J Immunol. (2012) 188:4951–8. 10.4049/jimmunol.1103160 22490869 PMC3345046

[25] Kobayashi KS van den Elsen PJ. NLRC5: a key regulator of MHC class I-dependent immune responses. Nat Rev Immunol. (2012) 12:813–20. 10.1038/nri3339 23175229

[26] Ludigs K Seguin-Estevez Q Lemeille S Ferrero I Rota G Chelbi S. NLRC5 exclusively transactivates MHC class I and related genes through a distinctive SXY module. PLoS Genet. (2015) 11:e1005088. 10.1371/journal.pgen.1005088 25811463 PMC4374748

[27] Gobin SJ Keijsers V van Zutphen M van den Elsen PJ. The role of enhancer A in the locus-specific transactivation of classical and nonclassical HLA class I genes by nuclear factor kappa B. J Immunol. (1998) 161:2276–83. 9725221

[28] Carey BS Poulton KV Poles A Factors affecting HLA expression: a review. Int J Immunogenet. (2019) 46:307–20. 10.1111/iji.12443 31183978

[29] Harton JA Zika E Ting JP The histone acetyltransferase domains of CREB-binding protein (CBP) and p300/CBP-associated factor are not necessary for cooperativity with the class II transactivator. J Biol Chem. (2001) 276:38715–20. 10.1074/jbc.M106652200 11514574

[30] Choi NM Majumder P Boss JM. Regulation of major histocompatibility complex class II genes. Curr Opin Immunol. (2011) 23:81–7. 10.1016/j.coi.2010.09.007 20970972 PMC3033992

[31] Morris AC Beresford GW Mooney MR Boss JM. Kinetics of a gamma interferon response: expression and assembly of CIITA promoter IV and inhibition by methylation. Mol Cell Biol. (2002) 22:4781–91. 10.1128/MCB.22.13.4781-4791.2002 12052885 PMC133907

[32] Devaiah BN Singer DS. CIITA and its dual roles in MHC gene transcription. Front Immunol. (2013) 4:476. 10.3389/fimmu.2013.00476 24391648 PMC3868913

[33] Vince N Li H Ramsuran V Naranbhai V Duh FM Fairfax BP. HLA-C level is regulated by a polymorphic Oct1 binding site in the HLA-C promoter region. Am J Hum Genet. (2016) 99:1353–8. 10.1016/j.ajhg.2016.09.023 27817866 PMC5142108

[34] Kaur G Gras S Mobbs JI Vivian JP Cortes A Barber T. Structural and regulatory diversity shape HLA-C protein expression levels. Nat Commun. (2017) 8:15924. 10.1038/ncomms15924 28649982 PMC5490200

[35] Ramsuran V Hernandez-Sanchez PG O'HUigin C Sharma G Spence N Augusto DG. Sequence and phylogenetic analysis of the untranslated promoter regions for HLA Class I genes. J Immunol. (2017) 198:2320–9. 10.4049/jimmunol.1601679 28148735 PMC5340644

[36] Hellweg CE Arenz A Bogner S Schmitz C Baumstark-Khan C. Activation of nuclear factor kappa B by different agents: influence of culture conditions in a cell-based assay. Ann N Y Acad Sci. (2006) 1091:191–204. 10.1196/annals.1378.066 17341614

[37] Li H Ivarsson MA Walker-Sperling VE Subleski J Johnson JK Wright PW. Identification of an elaborate NK-specific system regulating HLA-C expression. PLoS Genet. (2018) 14:e1007163. 10.1371/journal.pgen.1007163 29329284 PMC5785035

[38] Anderson SK. Molecular evolution of elements controlling HLA-C expression: adaptation to a role as a killer-cell immunoglobulin-like receptor ligand regulating natural killer cell function. HLA. (2018) 92:271–8. 10.1111/tan.13396 30232844 PMC6251751

[39] Johnson JK Wright PW Li H Anderson SK. Identification of trophoblast-specific elements in the HLA-C core promoter. HLA. (2018) 92:288–97. 10.1111/tan.13404 30270560 PMC6251741

[40] Ferreira LM Meissner TB Mikkelsen TS Mallard W O'Donnell CW Tilburgs T. A distant trophoblast-specific enhancer controls HLA-G expression at the maternal-fetal interface. Proc Natl Acad Sci USA. (2016) 113:5364–9. 10.1073/pnas.1602886113 27078102 PMC4868469

[41] Williams KL Taxman DJ Linhoff MW Reed W Ting JP. Cutting edge: Monarch-1: a pyrin/nucleotide-binding domain/leucine-rich repeat protein that controls classical and nonclassical MHC class I genes. J Immunol. (2003) 170:5354–8. 10.4049/jimmunol.170.11.5354 12759408

[42] Kufer TA Sansonetti PJ NLR functions beyond pathogen recognition. Nat Immunol. (2011) 12:121–8. 10.1038/ni.1985 21245903

[43] Sebastiano V Dalvai M Gentile L Schubart K Sutter J Wu GM. Oct1 regulates trophoblast development during early mouse embryogenesis. Development. (2010) 137:3551–60. 10.1242/dev.047027 20876643

[44] Clop A Bertoni A Spain SL Simpson MA Pullabhatla V Tonda R. An in-depth characterization of the major psoriasis susceptibility locus identifies candidate susceptibility alleles within an HLA-C enhancer element. PLoS ONE. (2013) 8:e71690. 10.1371/journal.pone.0071690 23990973 PMC3747202

[45] Kulkarni S Savan R Qi Y Gao X Yuki Y Bass SE. Differential microRNA regulation of HLA-C expression and its association with HIV control. Nature. (2011) 472:495–8. 10.1038/nature09914 21499264 PMC3084326

[46] O'Huigin C Kulkarni S Xu Y Deng Z Kidd J Kidd K. The molecular origin and consequences of escape from miRNA regulation by HLA-C alleles. Am J Hum Genet. (2011) 89:424–31. 10.1016/j.ajhg.2011.07.024 21907013 PMC3169826

[47] Tilburgs T Roelen DL van der Mast BJ de Groot-Swings GM Kleijburg C Scherjon SA. Evidence for a selective migration of fetus-specific CD4^+^CD25^bright^ regulatory T cells from the peripheral blood to the decidua in human pregnancy. J Immunol. (2008) 180:5737–45. 10.4049/jimmunol.180.8.5737 18390759

[48] Bulmer JN Williams PJ Lash GE. Immune cells in the placental bed. Int J Dev Biol. (2010) 54:281–94. 10.1387/ijdb.082763jb 19876837

[49] Faas MM de Vos P. Uterine NK cells and macrophages in pregnancy. Placenta. (2017) 56:44–52. 10.1016/j.placenta.2017.03.001 28284455

[50] Colonna M Borsellino G Falco M Ferrara GB Strominger JL. HLA-C is the inhibitory ligand that determines dominant resistance to lysis by NK1- and NK2-specific natural killer cells. Proc Natl Acad Sci USA. (1993) 90:12000–4. 10.1073/pnas.90.24.12000 8265660 PMC48113

[51] Lanier LL. NK cell receptors. Annu Rev Immunol. (1998) 16:359–93. 10.1146/annurev.immunol.16.1.359 9597134

[52] Parham P Moffett A. Variable NK cell receptors and their MHC class I ligands in immunity, reproduction and human evolution. Nat Rev Immunol. (2013) 13:133–44. 10.1038/nri3370 23334245 PMC3956658

[53] Middleton D Gonzelez F. The extensive polymorphism of KIR genes. Immunology. (2010) 129:8–19. 10.1111/j.1365-2567.2009.03208.x 20028428 PMC2807482

[54] Sharkey AM Gardner L Hiby S Farrell L Apps R Masters L. Killer Ig-like receptor expression in uterine NK cells is biased toward recognition of HLA-C and alters with gestational age. J Immunol. (2008) 181:39–46. 10.4049/jimmunol.181.1.39 18566368

[55] Hiby SE Walker JJ O'Shaughnessy KM Redman CW Carrington M Trowsdale J. Combinations of maternal KIR and fetal HLA-C genes influence the risk of preeclampsia and reproductive success. J Exp Med. (2004) 200:957–65. 10.1084/jem.20041214 15477349 PMC2211839

[56] Xiong S Sharkey AM Kennedy PR Gardner L Farrell LE Chazara O. Maternal uterine NK cell-activating receptor KIR2DS1 enhances placentation. J Clin Invest. (2013) 123:4264–72. 10.1172/JCI68991 24091323 PMC4382274

[57] Saito S Takeda Y Sakai M Nakabayahi M Hayakawa S. The incidence of pre-eclampsia among couples consisting of Japanese women and Caucasian men. J Reprod Immunol. (2006) 70:93–8. 10.1016/j.jri.2005.12.005 16427138

[58] Larsen TG Hackmon R Geraghty DE Hviid TVF. Fetal human leukocyte antigen-C and maternal killer-cell immunoglobulin-like receptors in cases of severe preeclampsia. Placenta. (2019) 75:27–33. 10.1016/j.placenta.2018.11.008 30712663

[59] Tilburgs T Crespo AC van der Zwan A Rybalov B Raj T Stranger B. Human HLA-G+ extravillous trophoblasts: Immune-activating cells that interact with decidual leukocytes. Proc Natl Acad Sci USA. (2015) 112:7219–24. 10.1073/pnas.1507977112 26015573 PMC4466754

[60] Tilburgs T Evans JH Crespo AC Strominger JL. The HLA-G cycle provides for both NK tolerance and immunity at the maternal-fetal interface. Proc Natl Acad Sci USA. (2015) 112:13312–7. 10.1073/pnas.1517724112 26460007 PMC4629323

[61] Blokhuis JH Hilton HG Guethlein LA Norman PJ Nemat-Gorgani N Nakimuli A. KIR2DS5 allotypes that recognize the C2 epitope of HLA-C are common among Africans and absent from Europeans. Immun Inflamm Dis. (2017) 5:461–8. 10.1002/iid3.178 28685972 PMC5691316

[62] Nakimuli A Chazara O Hiby SE Farrell L Tukwasibwe S Jayaraman J A KIR B centromeric region present in Africans but not Europeans protects pregnant women from pre-eclampsia. Proc Natl Acad Sci USA. (2015) 112:845–50. 10.1073/pnas.1413453112 25561558 PMC4311823

[63] Hiby SE Apps R Chazara O Farrell LE Magnus P Trogstad L. Maternal KIR in combination with paternal HLA-C2 regulate human birth weight. J Immunol. (2014) 192:5069–73. 10.4049/jimmunol.1400577 24778445 PMC4028203

[64] Jauniaux E Burton GJ. Pathophysiology of placenta accreta spectrum disorders: a review of current findings. Clin Obstet Gynecol. (2018) 61:743–54. 10.1097/GRF.0000000000000392 30299280

[65] Tilburgs T Strominger JL. CD8^+^ effector T cells at the fetal-maternal interface, balancing fetal tolerance and antiviral immunity. Am J Reprod Immunol. (2013) 69:395–407. 10.1111/aji.12094 23432707 PMC3711858

[66] Heemskerk MB Roelen DL Dankers MK van Rood JJ Claas FH Doxiadis II Allogeneic MHC class I molecules with numerous sequence differences do not elicit a CTL response. Hum Immunol. (2005) 66:969–76. 10.1016/j.humimm.2005.06.007 16360836

[67] Rudolph MG Wilson IA. The specificity of TCR/pMHC interaction. Curr Opin Immunol. (2002) 14:52–65. 10.1016/S0952-7915(01)00298-9 11790533

[68] Chien YH Davis MM. How alpha beta T-cell receptors ‘see' peptide/MHC complexes. Immunol Today. (1992) 14:597–602. 10.1016/0167-5699(93)90199-U 8305132

[69] Petersdorf EW Longton GM Anasetti C Mickelson EM McKinney SK Smith AG. Association of HLA-C disparity with graft failure after marrow transplantation from unrelated donors. Blood. (1997) 89:1818–23. 10.1182/blood.V89.5.1818.1818_1818_1823 9057668

[70] Meuleman T van Beelen E Kaaja RJ van Lith JM Claas FH Bloemenkamp KW HLA-C antibodies in women with recurrent miscarriage suggests that antibody mediated rejection is one of the mechanisms leading to recurrent miscarriage. J Reprod Immunol. (2016) 116:28–34. 10.1016/j.jri.2016.03.003 27172837

[71] Lissauer D Piper K Goodyear O Kilby MD Moss PA. Fetal-specific CD8^+^ cytotoxic T cell responses develop during normal human pregnancy and exhibit broad functional capacity. J Immunol. (2012) 189:1072–80. 10.4049/jimmunol.1200544 22685312

[72] van Kampen CA Versteeg-van der Voort Maarschalk MF Langerak-Langerak J van Beelen E Roelen DL Claas FH. Pregnancy can induce long-persisting primed CTLs specific for inherited paternal HLA antigens. Hum Immunol. (2001) 62:201–7. 10.1016/S0198-8859(01)00209-9 11250037

[73] Verdijk RM Kloosterman A Pool J van de KM Naipal AM van Halteren AG. Pregnancy induces minor histocompatibility antigen-specific cytotoxic T cells: implications for stem cell transplantation and immunotherapy. Blood. (2004) 103:1961–4. 10.1182/blood-2003-05-1625 14592836

[74] Tilburgs T Scherjon SA van der Mast BJ Haasnoot GW Versteeg-van der Voort Maarschalk M Roelen DL. Fetal-maternal HLA-C mismatch is associated with decidual T cell activation and induction of functional T regulatory cells. J Reprod Immunol. (2009) 82:147–56. 10.1016/j.jri.2009.05.003 19631389

[75] Erlebacher A. Immunology of the maternal-fetal interface. Annu Rev Immunol. (2013) 31:387–411. 10.1146/annurev-immunol-032712-100003 23298207

[76] Tilburgs T Schonkeren D Eikmans M Nagtzaam NM Datema G Swings GM. Human decidual tissue contains differentiated CD8^+^ effector-memory T cells with unique properties. J Immunol. (2010) 185:4470–7. 10.4049/jimmunol.0903597 20817873

[77] van der Zwan A Bi K Norwitz ER Crespo AC Claas FHJ Strominger JL. Mixed signature of activation and dysfunction allows human decidual CD8(+) T cells to provide both tolerance and immunity. Proc Natl Acad Sci USA. (2018) 115:385–90. 10.1073/pnas.1713957115 29259116 PMC5777048

[78] Tafuri A Alferink J Moller P Hammerling GJ Arnold B. T cell awareness of paternal alloantigens during pregnancy. Science. (1995) 270:630–3. 10.1126/science.270.5236.630 7570020

[79] Erlebacher A Vencato D Price KA Zhang D Glimcher LH. Constraints in antigen presentation severely restrict T cell recognition of the allogeneic fetus. J Clin Invest. (2007) 117:1399–411. 10.1172/JCI28214 17446933 PMC1849983

[80] Aluvihare VR Kallikourdis M Betz AG. Regulatory T cells mediate maternal tolerance to the fetus. Nat Immunol. (2004) 5:266–71. 10.1038/ni1037 14758358

[81] Xin L Ertelt JM Rowe JH Jiang TT Kinder JM Chaturvedi V Cutting edge: committed Th1 CD4^+^ T cell differentiation blocks pregnancy-induced Foxp3 expression with antigen-specific fetal loss. J Immunol. (2014) 192:2970–4. 10.4049/jimmunol.1302678 24591368 PMC3972488

[82] Zenclussen AC Gerlof K Zenclussen ML Sollwedel A Bertoja AZ Ritter T. Abnormal T-cell reactivity against paternal antigens in spontaneous abortion: adoptive transfer of pregnancy-induced CD4^+^CD25^+^ T regulatory cells prevents fetal rejection in a murine abortion model. Am J Pathol. (2005) 166:811–22. 10.1016/S0002-9440(10)62302-4 15743793 PMC1602357

[83] Tilburgs T Roelen DL van der Mast BJ van Schip JJ Kleijburg C de Groot-Swings GM Differential distribution of CD4^+^CD25^bright^ and CD8^+^CD28^−^ T-cells in decidua and maternal blood during human pregnancy. Placenta. (2006) 27:47–53. 10.1016/j.placenta.2005.11.008 16442616

[84] Mjosberg J Berg G Jenmalm MC Ernerudh J. FOXP3^+^ regulatory T cells and T helper 1, T helper 2, and T helper 17 cells in human early pregnancy decidua. Biol Reprod. (2010) 82:698–705. 10.1095/biolreprod.109.081208 20018909

[85] Sasaki Y Sakai M Miyazaki S Higuma S Shiozaki A Saito S. Decidual and peripheral blood CD4^+^CD25^+^ regulatory T cells in early pregnancy subjects and spontaneous abortion cases. Mol Hum Reprod. (2004) 10:347–53. 10.1093/molehr/gah044 14997000

[86] Du MR Guo PF Piao HL Wang SC Sun C Jin LP. Embryonic trophoblasts induce decidual regulatory T cell differentiation and maternal-fetal tolerance through thymic stromal lymphopoietin instructing dendritic cells. J Immunol. (2014) 192:1502–11. 10.4049/jimmunol.1203425 24453244 PMC3918863

[87] Svensson-Arvelund J Mehta RB Lindau R Mirrasekhian E Rodriguez-Martinez H Berg G. The human fetal placenta promotes tolerance against the semiallogeneic fetus by inducing regulatory T cells and homeostatic M2 macrophages. J Immunol. (2015) 194:1534–44. 10.4049/jimmunol.1401536 25560409

[88] Salvany-Celades M van der Zwan A Benner M Setrajcic-Dragos V Bougleux Gomes HA Iyer V Three types of functional regulatory T cells control T cell responses at the human maternal-fetal interface. Cell Rep. (2019) 27:2537–47 e5. 10.1016/j.celrep.2019.04.109 31141680

[89] Thornton AM Korty PE Tran DQ Wohlfert EA Murray PE Belkaid Y. Expression of Helios, an Ikaros transcription factor family member, differentiates thymic-derived from peripherally induced Foxp3^+^ T regulatory cells. J Immunol. (2010) 184:3433–41. 10.4049/jimmunol.0904028 20181882 PMC3725574

[90] Himmel ME MacDonald KG Garcia RV Steiner TS Levings MK Helios^+^ and Helios^−^ cells coexist within the natural FOXP3^+^ T regulatory cell subset in humans. J Immunol. (2013) 190:2001–8. 10.4049/jimmunol.1201379 23359504

[91] Tsuda S Zhang X Hamana H Shima T Ushijima A Tsuda K Clonally expanded decidual effector regulatory T cells increase in late gestation of normal pregnancy, but not in preeclampsia, in humans. Front Immunol. (2018) 9:1934 10.3389/fimmu.2018.01934 30197648 PMC6118230

[92] Inada K Shima T Ito M Ushijima A Saito S. Helios-positive functional regulatory T cells are decreased in decidua of miscarriage cases with normal fetal chromosomal content. J Reprod Immunol. (2015) 107:10–9. 10.1016/j.jri.2014.09.053 25453751

[93] Inada K Shima T Nakashima A Aoki K Ito M Saito S Characterization of regulatory T cells in decidua of miscarriage cases with abnormal or normal fetal chromosomal content. J Reprod Immunol. (2013) 97:104–11. 10.1016/j.jri.2012.12.001 23432877

[94] Steinborn A Haensch GM Mahnke K Schmitt E Toermer A Meuer S. Distinct subsets of regulatory T cells during pregnancy: is the imbalance of these subsets involved in the pathogenesis of preeclampsia? Clin Immunol. (2008) 129:401–12. 10.1016/j.clim.2008.07.032 18805741

[95] Wagner MI Jost M Spratte J Schaier M Mahnke K Meuer S Differentiation of ICOS^+^ and ICOS^−^ recent thymic emigrant regulatory T cells (RTE T regs) during normal pregnancy, pre-eclampsia and HELLP syndrome. Clin Exp Immunol. (2016) 183:129–42. 10.1111/cei.12693 26285098 PMC4687511

[96] Zhang Y Liu Z Tian M Hu X Wang L Ji J. The altered PD-1/PD-L1 pathway delivers the ‘one-two punch' effects to promote the Treg/Th17 imbalance in pre-eclampsia. Cell Mol Immunol. (2018) 15:710–23. 10.1038/cmi.2017.70 28890543 PMC6123412

[97] Crespo AC van der Zwan A Ramalho-Santos J Strominger JL Tilburgs T. Cytotoxic potential of decidual NK cells and CD8^+^ T cells awakened by infections. J Reprod Immunol. (2017) 119:85–90. 10.1016/j.jri.2016.08.001 27523927 PMC5290261

[98] Rasmussen SA Jamieson DJ Uyeki TM. Effects of influenza on pregnant women and infants. Am J Obstet Gynecol. (2012) 207:S3–8. 10.1016/j.ajog.2012.06.068 22920056

[99] Kenneson A Cannon MJ. Review and meta-analysis of the epidemiology of congenital cytomegalovirus (CMV) infection. Rev Med Virol. (2007) 17:253–76. 10.1002/rmv.535 17579921

[100] McDonagh S Maidji E Ma W Chang HT Fisher S Pereira L. Viral and bacterial pathogens at the maternal-fetal interface. J Infect Dis. (2004) 190:826–34. 10.1086/422330 15272412

[101] Goldenberg RL McClure EM Saleem S Reddy UM. Infection-related stillbirths. Lancet. (2010) 375:1482–90. 10.1016/S0140-6736(09)61712-8 20223514 PMC3893931

[102] Kuperman AA Zimmerman A Hamadia S Ziv O Gurevich V Fichtman B. Deep microbial analysis of multiple placentas shows no evidence for a placental microbiome. BJOG. (2019). 10.1111/1471-0528.15896. [Epub ahead of print]. 31376240

[103] de Goffau MC Lager S Sovio U Gaccioli F Cook E Peacock SJ. Human placenta has no microbiome but can contain potential pathogens. Nature. (2019) 572:329–34. 10.1038/s41586-019-1451-5 31367035 PMC6697540

[104] Pereira L. Congenital viral infection: traversing the uterine-placental interface. Annu Rev Virol. (2018) 5:273–99. 10.1146/annurev-virology-092917-043236 30048217

[105] El Costa H Gouilly J Mansuy JM Chen Q Levy C Cartron G. ZIKA virus reveals broad tissue and cell tropism during the first trimester of pregnancy. Sci Rep. (2016) 6:35296. 10.1038/srep35296 27759009 PMC5069472

[106] Zeldovich VB Robbins JR Kapidzic M Lauer P Bakardjiev AI. Invasive extravillous trophoblasts restrict intracellular growth and spread of Listeria monocytogenes. PLoS Pathog. (2011) 7:e1002005. 10.1371/journal.ppat.1002005 21408203 PMC3048367

[107] Adams Waldorf KM McAdams RM. Influence of infection during pregnancy on fetal development. Reproduction. (2013) 146:R151–62. 10.1530/REP-13-0232 23884862 PMC4060827

[108] Tabata T Petitt M Fang-Hoover J Pereira L. Survey of cellular immune responses to human cytomegalovirus infection in the microenvironment of the uterine-placental interface. Med Microbiol Immunol. (2019) 208:475–85. 10.1007/s00430-019-00613-w 31065796 PMC6635015

[109] Pereira L Maidji E. Cytomegalovirus infection in the human placenta: maternal immunity and developmentally regulated receptors on trophoblasts converge. Curr Top Microbiol Immunol. (2008) 325:383–95. 10.1007/978-3-540-77349-8_21 18637517

[110] Giugliano S Petroff MG Warren BD Jasti S Linscheid C Ward A. Hepatitis C virus sensing by human trophoblasts induces innate immune responses and recruitment of maternal NK cells: potential implications for limiting vertical transmission. J Immunol. (2015) 195:3737–47. 10.4049/jimmunol.1500409 26342030 PMC4592818

[111] Tuominen H Rautava S Syrjanen S Collado MC Rautava J. HPV infection and bacterial microbiota in the placenta, uterine cervix and oral mucosa. Sci Rep. (2018) 8:9787. 10.1038/s41598-018-27980-3 29955075 PMC6023934

[112] Pereira L Petitt M Fong A Tsuge M Tabata T Fang-Hoover J. Intrauterine growth restriction caused by underlying congenital cytomegalovirus infection. J Infect Dis. (2014) 209:1573–84. 10.1093/infdis/jiu019 24403553 PMC3997585

[113] Nigro G Mazzocco M Mattia E Di Renzo GC Carta G Anceschi MM. Role of the infections in recurrent spontaneous abortion. J Matern Fetal Neonatal Med. (2011) 24:983–9. 10.3109/14767058.2010.547963 21261443

[114] Racicot K Mor G. Risks associated with viral infections during pregnancy. J Clin Invest. (2017) 127:1591–9. 10.1172/JCI87490 28459427 PMC5409792

[115] Goldenberg RL Hauth JC Andrews WW. Intrauterine infection and preterm delivery. N Engl J Med. (2000) 342:1500–7. 10.1056/NEJM200005183422007 10816189

[116] Xie F Hu Y Magee LA Money DM Patrick DM Krajden M, Toxemia Study Group. An association between cytomegalovirus infection and pre-eclampsia: a case-control study and data synthesis. Acta Obstet Gynecol Scand. (2010) 89:1162–7. 10.3109/00016349.2010.499449 20804342

[117] Stern M Elsasser H Honger G Steiger J Schaub S Hess C. The number of activating KIR genes inversely correlates with the rate of CMV infection/reactivation in kidney transplant recipients. Am J Transplant. (2008) 8:1312–7. 10.1111/j.1600-6143.2008.02242.x 18444913

[118] Cook M Briggs D Craddock C Mahendra P Milligan D Fegan C. Donor KIR genotype has a major influence on the rate of cytomegalovirus reactivation following T-cell replete stem cell transplantation. Blood. (2006) 107:1230–2. 10.1182/blood-2005-03-1039 16239436

[119] Garcia-Beltran WF Holzemer A Martrus G Chung AW Pacheco Y Simoneau CR. Open conformers of HLA-F are high-affinity ligands of the activating NK-cell receptor KIR3DS1. Nat Immunol. (2016) 17:1067–74. 10.1038/ni.3513 27455421 PMC4992421

[120] De Re V Caggiari L De Zorzi M Repetto O Zignego AL Izzo F Genetic diversity of the KIR/HLA system and susceptibility to hepatitis C virus-related diseases. PLoS ONE. (2015) 10:e0117420 10.1371/journal.pone.0117420 25700262 PMC4336327

[121] Bonagura VR Du Z Ashouri E Luo L Hatam LJ DeVoti JA. Activating killer cell immunoglobulin-like receptors 3DS1 and 2DS1 protect against developing the severe form of recurrent respiratory papillomatosis. Hum Immunol. (2010) 71:212–9. 10.1016/j.humimm.2009.10.009 19861144 PMC2815039

[122] Crespo AC Strominger JL Tilburgs T. Expression of KIR2DS1 by decidual natural killer cells increases their ability to control placental HCMV infection. Proc Natl Acad Sci USA. (2016) 113:15072–7. 10.1073/pnas.1617927114 27956621 PMC5206558

[123] Chapel A Garcia-Beltran WF Holzemer A Ziegler M Lunemann S Martrus G. Peptide-specific engagement of the activating NK cell receptor KIR2DS1. Sci Rep. (2017) 7:2414. 10.1038/s41598-017-02449-x 28546555 PMC5445099

[124] van der Ploeg K Chang C Ivarsson MA Moffett A Wills MR Trowsdale J Modulation of human leukocyte antigen-C by human cytomegalovirus stimulates KIR2DS1 recognition by natural killer cells. Front Immunol. (2017) 8:298 10.3389/fimmu.2017.00298 28424684 PMC5372792

[125] Sim MJW Rajagopalan S Altmann DM Boyton RJ Sun PD Long EO. Human NK cell receptor KIR2DS4 detects a conserved bacterial epitope presented by HLA-C. Proc Natl Acad Sci USA. (2019) 116:12964–73. 10.1073/pnas.1903781116 31138701 PMC6601252

[126] Naiyer MM Cassidy SA Magri A Cowton V Chen K Mansour S. KIR2DS2 recognizes conserved peptides derived from viral helicases in the context of HLA-C. Sci Immunol. (2017) 2:eaal5296. 10.1126/sciimmunol.aal5296 28916719

[127] Dring MM Morrison MH McSharry BP Guinan KJ Hagan R Irish HCVRC. Innate immune genes synergize to predict increased risk of chronic disease in hepatitis C virus infection. Proc Natl Acad Sci USA. (2011) 108:5736–41. 10.1073/pnas.1016358108 21402922 PMC3078345

[128] Khakoo SI Thio CL Martin MP Brooks CR Gao X Astemborski J. HLA and NK cell inhibitory receptor genes in resolving hepatitis C virus infection. Science. (2004) 305:872–4. 10.1126/science.1097670 15297676

[129] Knapp S Warshow U Hegazy D Brackenbury L Guha IN Fowell A. Consistent beneficial effects of killer cell immunoglobulin-like receptor 2DL3 and group 1 human leukocyte antigen-C following exposure to hepatitis C virus. Hepatology. (2010) 51:1168–75. 10.1002/hep.23477 20077564 PMC4202114

[130] Thoens C Berger C Trippler M Siemann H Lutterbeck M Broering R KIR2DL3(+)NKG2A(-) natural killer cells are associated with protection from productive hepatitis C virus infection in people who inject drugs. J Hepatol. (2014) 61:475–81. 10.1016/j.jhep.2014.04.020 24780303

[131] La D Czarnecki C El-Gabalawy H Kumar A Meyers AF Bastien N. Enrichment of variations in KIR3DL1/S1 and KIR2DL2/L3 among H1N1/09 ICU patients: an exploratory study. PLoS ONE. (2011) 6:e29200. 10.1371/journal.pone.0029200 22216211 PMC3247251

[132] Rajagopalan S Fu J Long EO Cutting edge: induction of IFN-gamma production but not cytotoxicity by the killer cell Ig-like receptor KIR2DL4 (CD158d) in resting NK cells. J Immunol. (2001) 167:1877–81. 10.4049/jimmunol.167.4.1877 11489965

[133] Long EO Kim HS Liu D Peterson ME Rajagopalan S. Controlling natural killer cell responses: integration of signals for activation and inhibition. Annu Rev Immunol. (2013) 31:227–58. 10.1146/annurev-immunol-020711-075005 23516982 PMC3868343

[134] Burian A Wang KL Finton KA Lee N Ishitani A Strong RK. HLA-F and MHC-I open conformers bind natural killer cell Ig-like receptor KIR3DS1. PLoS ONE. (2016) 11:e0163297. 10.1371/journal.pone.0163297 27649529 PMC5029895

[135] Gamliel M Goldman-Wohl D Isaacson B Gur C Stein N Yamin R. Trained memory of human uterine NK cells enhances their function in subsequent pregnancies. Immunity. (2018) 48:951–62 e5. 10.1016/j.immuni.2018.03.030 29768178

[136] Liu D Tian S Zhang K Xiong W Lubaki NM Chen Z. Chimeric antigen receptor (CAR)-modified natural killer cell-based immunotherapy and immunological synapse formation in cancer and HIV. Protein Cell. (2017) 8:861–77. 10.1007/s13238-017-0415-5 28488245 PMC5712291

[137] Muntasell A Ochoa MC Cordeiro L Berraondo P Lopez-Diaz de Cerio A Cabo M. Targeting NK-cell checkpoints for cancer immunotherapy. Curr Opin Immunol. (2017) 45:73–81. 10.1016/j.coi.2017.01.003 28236750

[138] Norwitz ER Robinson JN Challis JR. The control of labor. N Engl J Med. (1999) 341:660–6. 10.1056/NEJM199908263410906 10460818

[139] Lissauer D Choudhary M Pachnio A Goodyear O Moss PA Kilby MD. Cytomegalovirus sero positivity dramatically alters the maternal CD8^+^ T cell repertoire and leads to the accumulation of highly differentiated memory cells during human pregnancy. Hum Reprod. (2011) 26:3355–65. 10.1093/humrep/der327 21979962

[140] Ameres S Mautner J Schlott F Neuenhahn M Busch DH Plachter B. Presentation of an immunodominant immediate-early CD8^+^ T cell epitope resists human cytomegalovirus immunoevasion. PLoS Pathog. (2013) 9:e1003383. 10.1371/journal.ppat.1003383 23717207 PMC3662661

[141] Makadzange AT Gillespie G Dong T Kiama P Bwayo J Kimani J. Characterization of an HLA-C-restricted CTL response in chronic HIV infection. Eur J Immunol. (2010) 40:1036–41. 10.1002/eji.200939634 20104487

[142] van Egmond A van der Keur C Swings GM Scherjon SA Claas FH The possible role of virus-specific CD8(+) memory T cells in decidual tissue. J Reprod Immunol. (2016) 113:1–8. 10.1016/j.jri.2015.09.073 26496155

[143] Gamadia LE Remmerswaal EB Surachno S Lardy NM Wertheim-van Dillen PM van Lier RA. Cross-reactivity of cytomegalovirus-specific CD8^+^ T cells to allo-major histocompatibility complex class I molecules. Transplantation. (2004) 77:1879–85. 10.1097/01.TP.0000131158.81346.64 15223907

[144] Amir AL D'Orsogna LJ Roelen DL van Loenen MM Hagedoorn RS de BR. Allo-HLA reactivity of virus-specific memory T cells is common. Blood. (2010) 115:3146–57. 10.1182/blood-2009-07-234906 20160165

[145] van der Zwan A van der Meer-Prins EMW van Miert P van den Heuvel H Anholts JDH Roelen DL. Cross-reactivity of virus-specific CD8^+^ T cells against allogeneic HLA-C: possible implications for pregnancy outcome. Front Immunol. (2018) 9:2880. 10.3389/fimmu.2018.02880 30574149 PMC6291497

[146] Chong AS Alegre ML Transplantation tolerance and its outcome during infections and inflammation. Immunol Rev. (2014) 258:80–101. 10.1111/imr.12147 24517427 PMC3927153

[147] Wang T Ahmed EB Chen L Xu J Tao J Wang CR. Infection with the intracellular bacterium, Listeria monocytogenes, overrides established tolerance in a mouse cardiac allograft model. Am J Transplant. (2010) 10:1524–33. 10.1111/j.1600-6143.2010.03066.x 20642679 PMC4060596

